# Burnout in medical students

**DOI:** 10.1007/s40211-020-00359-5

**Published:** 2020-09-03

**Authors:** L. Thun-Hohenstein, C. Höbinger-Ablasser, S. Geyerhofer, K. Lampert, M. Schreuer, C. Fritz

**Affiliations:** 1 Department for Child and Adolescent Psychiatry, Christian-Doppler Klinik (CDK), Salzburger Landeskrankenhaus (SALK), Paracelsus Private Medical University, Ignaz Harrerstsr. 79, 5020 Salzburg, Austria; 2 Department of Psychology, Webster University, Vienna, Austria; 3 Institute for Psychology, SALK, CDK, PMU, Salzburg, Austria; 4 Office for statistics Schreuer, Salzburg, Austria

**Keywords:** Mental health, Exhaustion, Areas of worklife, Stress, Medical education, Psychische Gesundheit, Erschöpfung, „Areas of worklife“, Stress, Medizinische Ausbildung

## Abstract

Only a small number of studies have examined the relationship between medical students and burnout syndrome. In Salzburg, Paracelsus Private Medical University (PMU) offers a 5‑year medical program instead of the regular 6 years of medical studies. Due to the tight schedule and heavy workload, the stress level of students is high. The purpose of this study was to determine whether PMU students show burnout symptoms. Three surveys were conducted: at the beginning of the academic year (T1, December 2009), at the end of the academic year (T2, June 2010), and at the beginning of the following academic year (T3, December 2010). For the assessment of burnout, the Maslach Burnout Inventory (emotional exhaustion, depersonalization or cynicism, and low personal accomplishment) was used, as well as the Six Factors Theory of Burnout (workload, control, reward, community, fairness, and values) and for comparison, the Austrian norms developed by Unterholzer. Burnout rate was calculated by a combined measure of the three components. The results show a significant difference from the norm means in emotional exhaustion, depersonalization/cynicism, and low personal accomplishment. With regard to areas of work life, all values are below the means, indicating high workload, high external control, low reward, low feeling of community, and low fairness—except values, i.e., motivation of the students. The mean overall burnout frequency turned out to be 47.8 ± 11.0%, whereas females have slightly higher burnout rates than males. An increasing linear trend with burnout rates was seen from the youngest to the oldest class. In addition, the estimated burnout rate increased within the academic term, as T2 had the highest rate, followed by T3, and the lowest rate was seen in T1. In conclusion, burnout in medical students is frequent and significantly related to heavy workload and other factors of worklife, necessitating changes of academic and organizational settings of medical curricula.

## Abstract (de)

Bisher gibt es nur wenige Studien zur Korrelation von Medizinstudierenden und Burnout-Syndrom. Die Paracelsus Medizinische Privatuniversität (PMU) in Salzburg offeriert ein fünfjähriges Medizinstudium anstatt der regulären Studienzeit von sechs Jahren. Das ist mit straffem Zeitplan und hoher Arbeitslast verbunden, weswegen auch das Stresslevel der Studierenden hoch ist. Um herauszufinden, ob die PMU-Studierenden Burnout-Symptome zeigen, wurden drei Befragungen durchgeführt. Die erste und zweite zu Beginn (T1, Dez 2009) bzw. gegen Ende (T2, Juni 2010) des akademischen Jahres, die dritte zu Beginn des darauffolgenden akademischen Jahres (T3, Dez 2010). Anhand des Maslach Burnout Inventory („Emotionale Erschöpfung“; „Depersonalisierung oder Zynismus“; „reduzierte persönliche Leistungsfähigkeit“) wurde die Burnout-Belastung gemessen und ein Burnout-Score errechnet. Um die Hypothese der Arbeitsplatzbelastungen als wesentlichen Auslösefaktor zu untersuchen, wurde die Six Factors Theory of Burnout (sog. „areas of worklife“, Bereiche des Arbeitslebens) ebenfalls untersucht. Die Werte wurden dann mit den von Unterholzer erstellten österreichischen Normen verglichen. Die Ergebnisse zeigten in den Bereichen „Emotionale Erschöpfung“, „Depersonalisierung oder Zynismus“ und „reduzierte persönliche Leistungsfähigkeit“ eine signifikante Erhöhung im Vergleich zu den Normwerten. In Bezug auf die „areas of worklife“ waren alle Werte außer dem der „values“, also der Motivation der Studierenden, unterdurchschnittlich, was auf hohe Arbeitslast, hohe externe Überwachung, wenig Belohnung, vermindertes Gruppengefühl und wenig Fairness hinweist. Es stellte sich heraus, dass die durchschnittliche Gesamt-Burnout-Rate bei 47,8 ± 11,0 % liegt, und somit im Bereich internationaler Studien. Weibliche Studierende waren etwas häufiger betroffen als männliche. Eine lineare Zunahme der Burnout-Rate über die Ausbildungsjahre hinweg ist zu erkennen. Außerdem ist die berechnete Burnout-Rate innerhalb eines akademischen Jahres ebenfalls angestiegen. Sie ist bei T2 am höchsten, gefolgt von T3, wobei T1 die Niedrigste aufweist. Zusammenfassend konnte eine deutlich erhöhte Rate an Burnout-Symptomen und ein signifikanter Zusammenhang mit dem „Arbeitsplatz“ Studium festgestellt. werden. Diese Resultate machen organisatorische und akademische Veränderung im medizinischen Curriculum nötig.

## Introduction

Good mental health and the absence of burnout are necessary for the development and maintenance of student and medical professionalism. Epidemiology of mental health data shows that more than 20% of medical students suffer from psychological disturbance and/or show mental health problems [1].

The Paracelsus Private Medical University (PMU) in Salzburg offers students a complete medical education within 5 years. Students are recruited after a standardized examination and interview process. Fifty students per year (out of about 600 applicants) had been accepted at the time of the study. During the last several years, and during the third year of study in particular, numerous students found their way to psychological or psychiatric services. These observations lead to the inception of this study about burnout in medical students.

Maslach et al. [2] defined burnout as a syndrome of exhaustion comprised of emotional exhaustion, depersonalization and low personal accomplishment. Clinical burnout—although not a clinical diagnosis—can be distinguished by high scores in emotional exhaustion or depersonalization and low personal accomplishment [3, 4]. According to Maslach and Leiter [5] burnout is the consequence of a mismatch between work place/surroundings and the working person; thus, in the case of medical students, burnout could be hypothesized to be a mismatch between the student and conditions at the university.

Studies about stress [6] and alcohol abuse [7] in medical students have been published since the early 1980s. Within the last decades, several studies have confirmed the high amount of psychosocial pressure and low social support of medical students, resulting in high rates of depression (12%) [8] and burnout (47% [9]; 45% [10]) [11, 12]. In a meta-analysis of studies published between 1984 and 2005, Dyrbye et al. [13] described that according to these studies medical students experience significantly more overall stress and consequently have increased rates of mental health problems. Dahlin et al. [8], for example, found 27% of students to have a psychiatric diagnosis, of whom only 4.2% sought help. More recent studies revealed similar results [14, 15], showing that emotional exhaustion and high cynicism indicate that students experience a high level of burnout.

Exhaustion is thought to appear as a combined symptom of the individual stress experience at the workplace. Job stress has been recognized as a central occupational contributor to personal stress experience; thus, the subjective internal stress level is believed to be the mediator between the impact of job demands and work-related outcomes. Central to this experience is the fact that the workload is exceeding personal working resources. Overwhelming workload is highly correlated with the exhaustion dimension of burnout [16]. Furthermore, exhaustion seems to have a mediating function for the other two dimensions of burnout. To counteract this disequilibrium the person needs to develop control to influence decisions concerning their work and their workload, which on the other hand is connected to a sense of efficacy.

A further dimension of workplace areas is the reward a person receives for their work, which can be monetary, social, or/and intrinsic. For medical students, monetary reward is not given but replaced by study results, intrinsic reward is a very personal factor—depending on interests, intellectual or learning capacity, thus social reward will be a very important factor of reward at universities. Insufficient reward increases the persons vulnerability to burnout [17]. Concerning the social dimension of workplace community factors as quality of interaction, conflict management, mutual support, closeness, and teamwork have a significant influence on burnout prevention or development [18].

Fairness—defined as the perception of being treated with respect and equally by the authorities—is significantly related to a person’s self-worth and—if missing—to the development of burnout [19]. The central factor of people’s relationship to work and/or workplace is the value area. Motivation is the central factor of good working experience and the connection between employer and employee.

Thus, the Areas of Worklife Survey (AWLS) covers several important issues connected to the development of burnout symptoms. This is the first study on student burnout to explore the Six-Factor Theory, assuming that to study at a university is comparable to a workplace situation.

Hence, we were interested in exploring whether our medical students have similar results regarding clinical burnout rates and whether student’s burnout is related to the Six Factors Theory of Burnout [3]. We expected high rates of burnout. Furthermore, we hypothesized that burnout in students is related to the high workload as a result of studying for exams as well as high stress levels.

## Methods

After approval by the Ethics Commission Salzburg (415-EP/65/3-2009), the study was performed in cooperation with Webster University, where the normative data collection for burnout in Austria was performed [20] and guaranteeing that the subjective data were not available to the authors working for PMU.

After students signed the informed consent, the individual assessment was performed via internet by means of a red-dot-programmed database. Personal results were sent to students via email from Webster University, thus giving the students the possibility to react to their results. In addition, they were offered professional help on a voluntary basis.

Assessment was performed at three points in time: at the beginning of the academic year (T1, December 2009), halfway through the academic year (T2, June 2010) and at the beginning of the following academic year (T3, December 2010). Due to insufficient student participation, surveys 2 and 3 were conducted in two waves, respectively. As results of the respective waves did not differ significantly concerning the confounding factors or main results, data are reported as surveys 1, 2, and 3.

## Material

The Maslach Burnout Inventory (MBI) [2] was used for the assessment of burnout. It consists of three subscales: emotional exhaustion (EE), depersonalization or cynicism (CY), and low (reduced) personal accomplishment (redPE). The reliability of the MBI expressed as Cronbach’s alpha is 0.89, 0.78, and 0.81 for the three subscales, respectively [21]. Predictive validity seems to be sufficient as well, as was demonstrated by Dyrbye and Shanafelt [22] and Dyrbye et al. [23]. In accordance with the Austrian normative data by Unterholzer [20], burnout will be defined as a combined measure and used in a dichotomized way (burnout yes = if EE >3.3 and CY >2.5 and redPE >1.9).

For assessment of the Six Factors Theory, a German version [24] of the Areas of Worklife Survey (AWLS [2]) was used. It consists of six subscales comprising six strategic areas (workload, control, reward, community, fairness, and values), which are part of the Six Factors Theory of burnout by Maslach and Leiter [3]. Each scale includes negatively and positively formulated items and participants indicate the degree of agreement by a 5-point Likert scale: 1 (strongly disagree)—3 (hard to decide)—5 (strongly agree). Scoring for negatively formulated items is reversed. For each scale, mismatch of person to job is indicated by a low score (less than 3) and a good match with a score greater than 3. Internal (Cronbach’s alpha for the subscales between “values” 0.73 to 0.84 “workload”) and external validity with the burnout scale MBI was found to be good [19].

## Statistics

Statistical analysis included descriptive statistics and comparison of the results to normative data by t‑test and z‑scores as well as group comparison statistics. Furthermore, a multivariate longitudinal analysis was applied. For influences of the different factors we used the Generalized Estimating Equations (GEE) analysis. We examined which factors have a significant effect on the prediction of the response variable (or outcome variable): burnout (yes, no). Data from all three surveys is included and the correlation of the responses (due to the fact that many subjects were included in all three surveys) is taken into account. Due to the dichotomous nature of the response variable, the model fitted will be a logistic regression model with correlated data. The statistical procedure to carry out this analysis is referred to as GEE with the logit link function. The correlation matrix structure chosen was “Compound Symmetry” (also referred to as “Exchangeable”). This correlation structure assumes that the responses from the three surveys are correlated pair-wise with the same correlation ρ. This correlation structure was chosen because it proved to be (slightly) better and moreover simpler than the general correlation matrix “Unstructured” which allows different correlations between the responses of the three surveys.

The “Factors” or confounding variables considered were “Survey No.”, “Gender”, “1st year at PMU”, “Relationship status”, “Nationality”, “Health” (Do you do anything to stay healthy or to improve your health? Answer categories: no, tried but gave up, yes) and “Domicile” (in/outside Salzburg Province).

## Subjects

The study was carried out between December 2009 and February 2011 and consisted of three surveys which took place roughly every 6 months, i.e, at the beginning of the academic year, halfway through and at the end of the academic year. Participation was voluntary and free. A total of *N* = 135/250 (54%) medical students signed the informed consent and participated—41 of them participated in all three surveys, 51 in two of the three surveys and 43 only once. Of the 135 students, 115 responded to the first survey, 16 responded for the first time to the second survey and 4 responded for the first time to the third survey. The total number of students participating in each of the three surveys was 115 for the first survey, 87 for the second survey and 66 for the third survey.

Of the 135 medical students, 57.8% were female and 42.2% male, further subdivided into their first year of study: 2006 (*n* = 28), 2007 (*N* = 28), 2008 (*n* = 39), and 2009 (*n* = 40) and nationality: Austrian (*n* = 94), German (*n* = 36), and others (*n* = 4). For survey 1 specifically, the total of 115 participants were subdivided into the following: 40.9% male, 59.1% female; of whom were 67.5% Austrian, 29.8% German, and 2.6% belonged to another nationality. The class distribution was as follows: 2006 (20.9%), 2007 (13.0%), 2008 (32.2%), and 2009 (33.9%), respectively. For survey 2, the total of 87 participants were subdivided into: 46.0% male, 54.0% female; of whom were 74.4% Austrian, 22.1% German, and 3.5% belonged to another nationality. The class distribution was as follows: 2006 (12.6%), 2007 (23.0%), 2008 (29.9%), and 2009 (34.5%), respectively. For survey 3, the total of 66 participants were subdivided into: 40.9% male, 59.1% female; of whom were 62.2% Austrian, 36.4% German, and 1.5% belonged to another nationality. The class distribution was as follows: 2006 (24.2%), 2007 (21.2%), 2008 (31.8%), and 2009 (22.7%), respectively.

The students were all of similar age at the first survey—mean age 21.5 years (standard deviation [SD] = 1.9), nearly 90% were between 20 and 25 years, ten 18- to 19-year-olds and four aged 26 to 28 years.

Other background variables surveyed were relationship status, place of residence, general fitness, and whether any action was taken to stay healthy or to improve one’s health. The answers are summarized as follows: concerning relationship status, 60% reported to be single, 40% in a relationship. The place of residence for 76–83% was within the province of Salzburg and 17–24% outside of Salzburg. General fitness was reported to be good by 77–83% of participants, 17–23% did not report good fitness. Concerning active health behavior (“Do you do anything to keep fit or to improve your health?”), roughly 80% reported to perform some, 6–8% did none, and 12–15% tried but gave up.

## Results

### Burnout rates

Results for the subscales of the MBI for each survey as well as in comparison to normative values from the Austrian study by Unterholzer [20] are provided in Table 1. For the subscale EE, the sample means differ significantly from the norm means in all three surveys (t-tests, in all cases *p* < 0.0005). For the subscale CY in survey 1 (2.51 ± 0.91), there is no significant difference in the first survey, in survey 2 (3.11 ± 1.26), and survey 3 (2.88 ± 1.21) the difference is significant (t-tests; *p* = 0.943, *p* < 0.0005 and *p* = 0.013, respectively). Subscale redPE: In all three surveys, the sample means (2.10 ± 0.63, 2.46 ± 0.72, and 2.36 ± 0.70) differ highly significantly from the norm mean (t-tests; *p* = 0.001, *p* < 0.0005, and *p* < 0.0005, respectively).

**Table 1:** Means and SDs and z‑scores of MBI subscales EE, CY and redPE by survey

	Norm sample (*N* = 868)		Survey 1 (*N* = 115)			Survey 2 (*N* = 87)			Survey 3 (*N* = 66)		
	Mean^a^	SD^a^	Mean	SD	Z‑score	Mean	SD	Z‑score	Mean	SD	Z‑score
EE	3.48	1.101	3.97^***^	0.898	0.45	4.55^***^	0.871	0.97	4.25^***^	1.023	0.70
CY	2.84	1.161	2.51^***,b^	0.915	−0.28	3.11^**^	1.257	0.23	2.88	1.210	0.03
redPE	1.97	0.628	2.10^*^	0.634	0.21	2.46^***^	0.723	0.78	2.36^***^	0.705	0.62

*MBI* Maslach Burnout Inventory, *EE* emotional exhaustion, *CY* Cynicism, *redPE* reduced personal accomplishment, *SD* standard deviation

^*^Weakly significant with 0.05 < *p* ≤ 0.10, ^**^significant with 0.01 < *p* ≤ 0.05, ^***^highly significant with *p* ≤ 0.01

^a^Values taken from Unterholzer 2008 [20, p. 126]

^b^Significantly *below* the norm-mean

With respect for survey 1, the combined burnout frequency for males was 25.5% (*N* = 47, *n* = 12), and 39.7% for females (*N* = 47, *n* = 12). In survey 2, the results were much higher with a combined burnout frequency of 52.5% in males (*N* = 40, *n* = 21) and 68.1% for females (*N* = 47, *n* = 32). In survey 3, however, the numbers decreased slightly to 40.7% in males (*N* = 27, *n* = 11), and to 53.8% for females (*N* = 39, *n* = 21). Concluding a total burnout frequency of 33.9%, 60.9%, and 48.5% for T1–3, respectively.

The calculation of the combined mean burnout measure demonstrated a mean overall burnout frequency in the whole sample of 47.8 ± 11.0%. Although females have higher burnout rates than males in all three surveys, this difference is not statistically significant in any of them (survey 1 *p* = 0.114; survey 2 *p* = 0.138; survey 3 *p* = 0.295, respectively); 24.4% never had elevated burnout rates.

### Areas of worklife

Table 2 presents the results of the AWLS subscales for each survey. With few exceptions, the sample means lie significantly below the norm means—in other words, on average there is low mean Workload (i.e., high workload), low mean Control (i.e., high external control), low mean Reward, low mean feeling of Community, low mean Fairness and low mean Values. The exceptions are: survey 1/Reward and survey 1/Fairness which, although slightly below the norm mean, do not differ significantly and survey 1/Values which is significantly higher than the norm mean. All values of all factors decrease at T2 and slightly increase at T3, but never reach a normative level.

**Table 2:** Areas of Worklife Survey (AWLS) subscales, means, standard deviations (SDs) and z‑scores per survey

	Norm sample		Survey 1 (*N* = 115)			Survey 2 (*N* = 87)			Survey 3 (*N* = 66)		
	Mean^a^	SD	Mean	SD	z‑score	Mean	SD	z‑score	Mean	SD	z‑score
(Low) Workload	3.76	1.02	2.74^***^	0.77	−1.32	2.48^***^	0.77	−1.66	2.64^***^	0.86	−1.30
Control	3.9	0.91	2.73^***^	0.89	−1.31	2.23^***^	0.93	−1.80	2.50^***^	0.97	−1.44
Reward	3.7	0.89	3.62	0.71	−0.07	3.28^***^	0.79	−0.53	3.42^***^	0.83	−0.34
Community	4	0.87	3.73^***^	0.84	−0.32	3.61^***^	0.89	−0.44	3.71^***^	0.78	−0.37
Fairness	3.4	0.88	3.35	0.72	−0.07	2.91^***^	0.76	−0.64	3.16^***^	0.66	−0.36
Values	3.5	0.75	3.69^***,b^	0.72	0.26	3.19^***^	0.76	−0.41	3.33^**^	0.66	−0.26

^*^Weakly significant with 0.05 < *p* ≤ 0.10, ^**^significant with 0.01 < *p* ≤ 0.05, ^***^highly significant with *p* ≤ 0.01

^a^Values taken from Unterholzer 2008 [20, p. 126]

^b^Significantly *above *the norm-mean; for all other comparisons the sample means lie below the norm means

### Correlations of burnout with AWLS

In Table 3 the correlations of the combined burnout rate and subscales of the AWLS subscales are presented and show a high correlation with all the subscales of the AWLS.

**Table 3:** Correlation of Areas of Worklife Survey (AWLS) subscales to combined burnout measures

			EE	CY	redPE	Burnout
Survey 1 *N* = 115	(Low) Workload	*r*	−0.736^**^	−0.472^**^	−0.348^**^	−0.381^**^
		*p*	<0.0005	<0.0005	<0.0005	<0.0005
	Control	*r*	−0.431^**^	−0.443^**^	−0.316^**^	−0.341^**^
		*p*	<0.0005	<0.0005	0.001	<0.0005
	Reward	*r*	−0.385^**^	−0.623^**^	−0.595^**^	−0.552^**^
		*p*	<0.0005	<0.0005	<0.0005	<0.0005
	Community	*r*	−0.101	−0.416^**^	−0.352^**^	−0.237^*^
		*p*	0.286	<0.0005	<0.0005	0.012
	Fairness	*r*	−0.320^**^	−0.524^**^	−0.369^**^	−0.353^**^
		*p*	0.001	<0.0005	<0.0005	<0.0005
	Values	*r*	−0.198^*^	−0.504^**^	−0.394^**^	−0.369^**^
		*p*	0.035	<0.0005	<0.0005	<0.0005
Survey 2 *N* = 87	(Low) Workload	*r*	−0.770^**^	−0.626^**^	−0.577^**^	−0.520^**^
		*p*	<0.0005	<0.0005	<0.0005	<0.0005
	Control	*r*	−0.527^**^	−0.573^**^	−0.479^**^	−0.437^**^
		*p*	<0.0005	<0.0005	<0.0005	<0.0005
	Reward	*r*	−0.575^**^	−0.638^**^	−0.739^**^	−0.486^**^
		*p*	<0.0005	<0.0005	<0.0005	<0.0005
	Community	*r*	−0.359^**^	−0.470^**^	−0.521^**^	−0.302^**^
		*p*	0.001	<0.0005	<0.0005	0.005
	Fairness	*r*	−0.550^**^	−0.555^**^	−0.539^**^	−0.440^**^
		*p*	<0.0005	<0.0005	<0.0005	<0.0005
	Values	*r*	−0.513^**^	−0.705^**^	−0.627^**^	−0.539^**^
		*p*	<0.0005	<0.0005	<0.0005	<0.0005
Survey 3 *N* = 66	(Low) Workload	*r*	−0.775^**^	−0.620^**^	−0.607^**^	−0.608^**^
		*p*	<0.0005	<0.0005	<0.0005	<0.0005
	Control	*r*	−0.521^**^	−0.430^**^	−0.533^**^	−0.386^**^
		*p*	<0.0005	<0.0005	<0.0005	0.001
	Reward	*r*	−0.327^**^	−0.498^**^	−0.540^**^	−0.375^**^
		*p*	0.008	<0.0005	<0.0005	0.002
	Community	*r*	−0.101	−0.383^**^	−0.652^**^	−0.253^*^
		*p*	0.425	0.002	<0.0005	0.042
	Fairness	*r*	−0.237	−0.394^**^	−0.453^**^	−0.337^**^
		*p*	0.058	0.001	<0.0005	0.006
	Values	*r*	−0.133	−0.425^**^	−0.532^**^	−0.252^*^
		*p*	0.292	<0.0005	<0.0005	0.043

*MBI* Maslach Burnout Inventory, *EE* emotional exhaustion, *CY* Cynicism, *redPE* reduced personal accomplishment, *SD* standard deviation

^**^Correlation is significant at the 0.01 level (2-tailed), ^*^Correlation is significant at the 0.05 level (2-tailed)

### Confounding factors

#### First year at PMU, gender, relationship status, health activity, and nationality and burnout

In order to control for some influential factors of burnout, we included background factors such as being a first year student, gender, relationship status, and health activity in our study. Table 4 presents burnout results as subscales of the MBI for survey and first year at PMU and significance levels.

**Table 4:** Means and SDs of MBI subscales EE, CY, and redPE by survey and first year at PMU and significance levels

			Survey 1			Survey 2			Survey 3		
			*N*	Mean	SD	*N*	Mean	SD	*N*	Mean	SD
EE	1st year	2009	39	3.61	1.02	30	4.19	0.87	15	3.76	0.89
		2008	37	4.22	0.75	26	4.58	0.62	21	4.77	0.84
		2007	15	4.12	0.86	20	5.26	0.71	14	4.19	0.88
		2006	24	4.08	0.77	11	4.15	0.92	16	4.07	1.24
		Total	115	3.97	0.90	87	4.55	0.87	66	4.25	1.02
		*p*	<0.0019			<0.005			<0.021		
CY	1st year	2009	39	2.21	0.93	30	2.75	1.21	15	2.27	1.07
		2008	37	2.51	0.80	26	2.82	0.83	21	3.06	1.04
		2007	15	2.44	0.81	20	4.08	1.30	14	2.70	1.11
		2006	24	3.03	0.93	11	3.05	1.39	16	3.39	1.43
		Total	115	2.51	0.91	87	3.11	1.26	66	2.88	1.21
		*p*	<0.006			<0.001			<0.056		
redPE	1st year	2009	39	2.13	0.62	30	2.44	0.86	15	2.36	0.76
		2008	37	2.05	0.46	26	2.38	0.49	21	2.45	0.57
		2007	15	2.03	0.79	20	2.78	0.71	14	2.15	0.82
		2006	24	2.18	0.80	11	2.15	0.68	16	2.42	0.73
		Total	115	2.10	0.63	87	2.46	0.72	66	2.36	0.70
		*p*	<0.828			0.103			0.657		

*MBI* Maslach Burnout Inventory, *EE* emotional exhaustion, *CY* Cynicism, *redPE* reduced personal accomplishment, *SD* standard deviation

#### First year at PMU

As can be seen the four student groups differ significantly with respect to meanEE and meanCY at all three points in time, but do not differ significantly with respect to mean redPE. A closer look at the data reveals the following pattern for survey 1:

- with respect to Emotional Exhaustion, it is the youngest class of students (2009) that has a significantly lower meanEE than the other three groups, which among themselves do not differ significantly;
- with respect to Cynicism, it is the oldest class of students (2006) that has a higher meanCY than the other three groups, which among themselves do not differ significantly.

For survey 2, it is the class of students that started in 2007 that has significantly higher meanEE and meanCY than the other three groups, which do not differ significantly among themselves. For survey 3 there is no clear pattern. The class that started in 2008 has the highest meanEE, significantly higher than 2009 (*p* = 0.003) and 2006 (*p* = 0.034) and weakly significantly higher than 2007 (*p* = 0.085). With respect to Cynicism it is again the class of 2009 that has the lowest meanCY, significantly different to 2008 (*p* = 0.049) and 2006 (*p* = 0.010) but not significantly different to 2007 (*p* = 0.378).

Table 5 shows the combined burnout rate and first year at PMU. In survey 1 the four burnout rates do not differ significantly from each other (Chi-squared test *p* = 0.280) but there is an (almost) significant linear trend (*p* = 0.058) with burnout rates increasing from 23.1% for the youngest class which started in 2009 to 45.8% for the oldest class which started in 2006. In survey 2, the four burnout rates differ significantly (Chi-squared test *p* = 0.032). The highest rate of 85% is for the class that started in 2007 (this class had the highest meanEE and meanCY in survey 2). In survey 3, the Chi-squared test comparing all four groups is weakly significant (*p* = 0.079).

**Table 5:** Combined burnout measures and first year at Paracelsus Private Medical University (PMU)

		Survey 1			Survey 2			Survey 3		
		*N*	Burnout *n*	Burnout %	*N*	Burnout *n*	Burnout %	*N*	Burnout *n*	Burnout %
1st year	2009	39	9	23.1	30	13	43.3	15	3	20.0
	2008	37	13	35.1	26	16	61.5	21	13	61.9
	2007	15	6	40.0	20	17	85.0	14	7	50.0
	2006	24	11	45.8	11	7	63.6	16	9	56.3
	Total	115	39	33.9	87	53	60.9	66	32	48.5

The burnout rate for the youngest students starting in 2009 was much lower than the rates of the other three groups (61.9%, 50.0%, and 56.3%) which do not differ significantly among themselves. For the pair-wise comparisons 2009 vs. 2008, 2009 vs. 2007, and 2009 vs. 2006, the *p*-values of the Chi-squared tests are *p* = 0.013, *p* = 0.089, and *p* = 0.038, respectively. In all three surveys, the burnout rates of the class of students that started in 2009 are the lowest.

#### Predictability of burnout

##### Results of calculation by the GEE model

Only the first four factors (“Survey No.”, “Gender”, “1st year at PMU”, “Family status”, “Nationality”, “Health” “Domicile”) proved statistically significant. The “Health” factor looked “interesting” and was almost weakly significant, but since the vast majority of the students answered “yes” and only few (5 to 14) “no” or “tried but gave up” this factor was no longer considered. The model included main effects only; an interaction effect “Gender” × “Family status” was examined, but was found to be not significant (*p* = 0.394).

##### Summary of the GEE model

- Dependent variable: burnout (yes/no)
- Link function: logit (logistic regression)
- Correlation structure: compound symmetry

##### Factors (predictor variables): survey no. (*p* < 0.0005), gender (*p* = 0.034), 1st year at PMU (*p* = 0.007), and family status (*p* = 0.033)

**Fig. 1:**
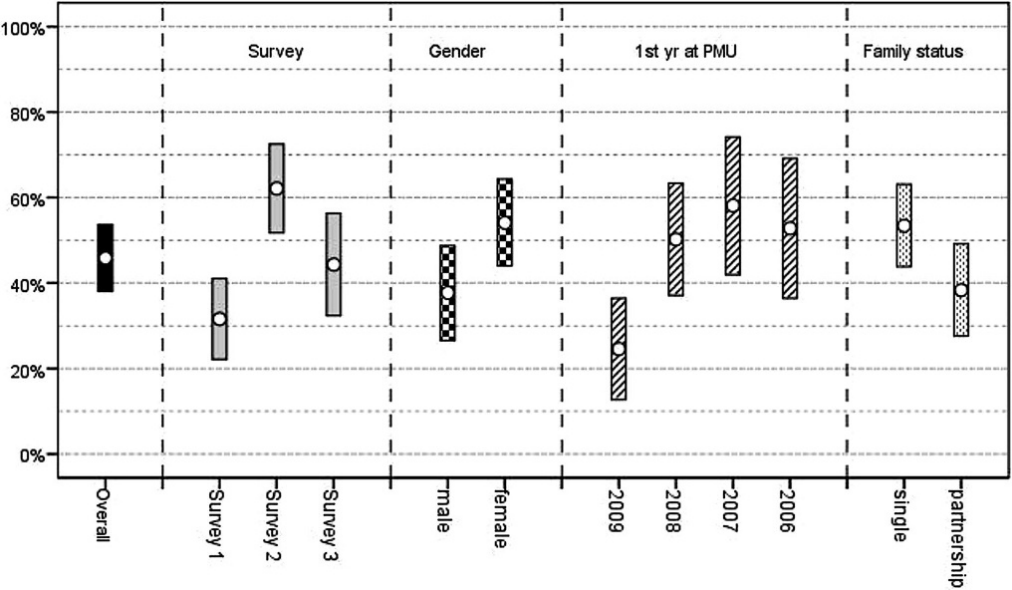
Predicted probability (+95%confidence interval) for Burnout (in %)—overall and in various subgroups. *yr* year, *PMU* Paracelsus Private Medical University

The fitted model enabled us to calculate the expected probability of burnout (+95% confidence intervals [CIs]) for every one of the 3 × 2 × 4 × 2 = 48 combinations of the predictors. By averaging over all these expected probabilities, one receives the overall expected probability for burnout (based on all the data of all three surveys). Likewise, in order to calculate the expected marginal probabilities for the categories of any one of the factors, one averages over all cells generated by the rest of the factors. For example, to get the marginal probabilities for survey 1, survey 2, and survey 3 one averages over the remaining 16 cells (Gender: 2 × 1st year: 4 × Family Status: 2) for each of the three surveys. The results are summarized in Fig. 1.

The effects of the four factors are summarised below.

#### Survey effect

From survey 1 to survey 2 the estimated burnout rate increases by 30.5% (*p* < 0.0005; 95% confidence interval [CI] 17.7% to 43.3%); from survey 2 to survey 3 the rate drops by 17.8% (*p* = 0.016; 95%CI 3.4% to 32.2%). The difference between survey 1 and survey 3 is an increase of 12.0% (*p* = 0.050; 95%CI 0% to 25.5%).

#### Gender effect

The estimated burnout rate for females is 16.5% higher (*p* = 0.031; 95%CI 1.5% to 31.4%) than the rate for males.

#### Year effect

The estimated burnout rate for students who started their studies in 2009 is significantly lower than the rates for the other three year groups: 25.7% lower than 2008 (*p* = 0.004; 95%CI 8.0% to 43.3%), 33.5% lower than 2007 (*p* = 0.001; 95%CI 13.8% to 53.3%), and 28.2% lower than 2006 (*p* = 0.006; 95%CI 8.0% to 48.4%). The year groups 2008, 2007, and 2006 do not differ significantly in pair-wise comparisons among themselves.

#### Family status effect

The estimated burnout rate among students in a relationship is 15.1% lower (*p* = 0.030; 95%CI 1.5% to 28.7%) than that of single students. A test of possible interaction between “Gender” and “Family status” proved to be not significant (*p* = 0.394).

**Fig. 2:**
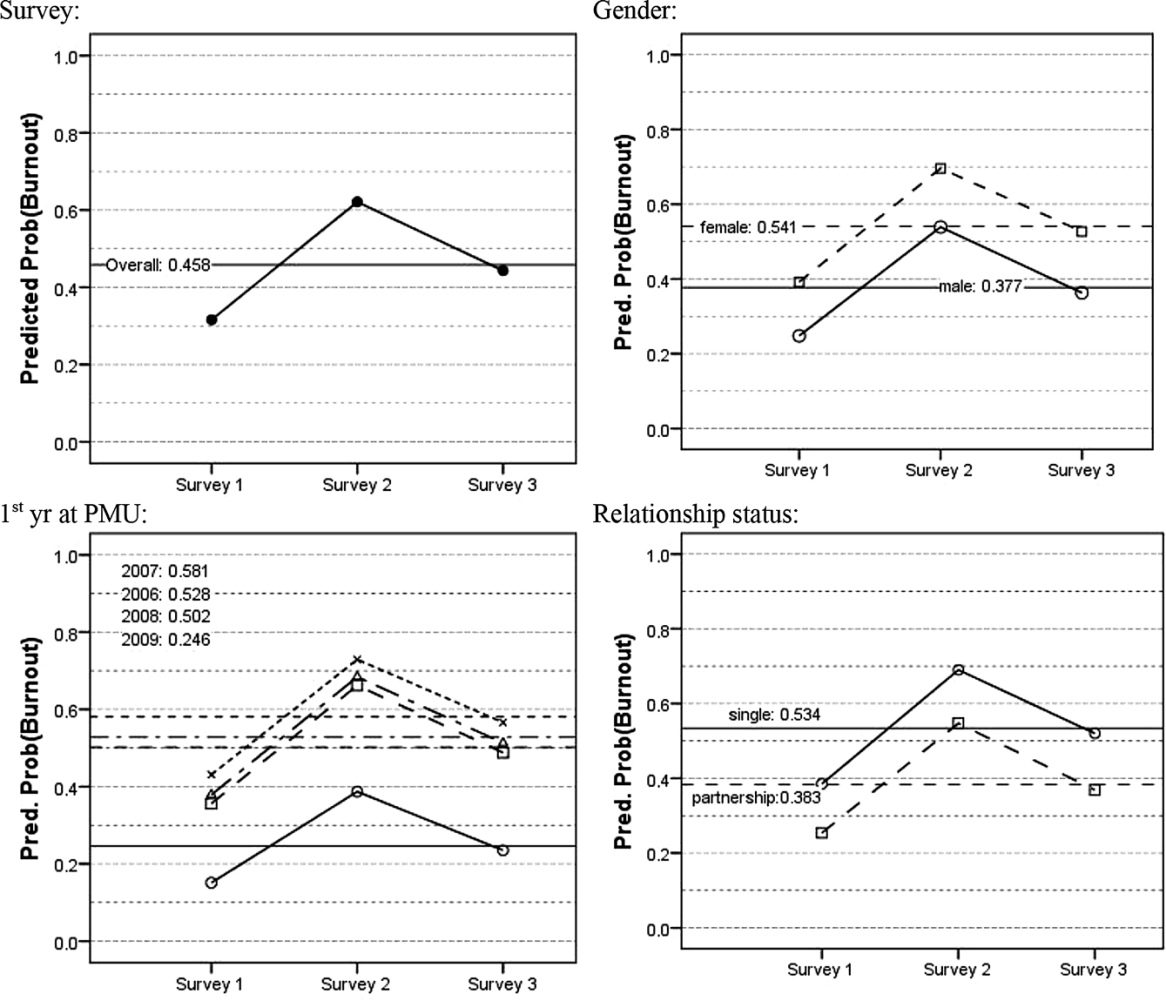
Predicted probability (Predict Prob) of Burnout (by survey and overall) for all factors in the model. *yr* year, *PMU* Paracelsus Private Medical University

Burnout rates were also calculated separately for Gender, 1st year at PMU and Family status for *each survey* separately. These are presented graphically as line diagrams in Fig. 2.

## Discussion

The main results of this study—a combined burnout rate of 49%—confirm the high rate of burnout (34.2% to 52.6%) in medical students found in several studies in the US [10, 13, 24–29] and Europe [28, 29]. Lower rates were reported in Brazil (10.3% [30], 26.4% [31], and 28% [32]) or Australia (6%) [33]. However, according to Frajerman et al., the worldwide burnout prevalence for medical students lies around 44% [34].

Burnout is considered a measure of professional distress in three domains: emotional exhaustion, depersonalization, and low sense of personal accomplishment. Emotional exhaustion is described as the feeling of being emotionally depleted by one’s work. All subscales of the MBI showed significant changes, meaning that students showed high levels of exhaustion, high cynicism and high depersonalization. Depersonalization is recognized by treating people as if they are impersonal objects and low sense of personal accomplishment is best described by feeling that one’s work is not important or inconsequential. Not only the burnout rate, which is a combined measure as described in the method section, but also the means of the MBI subscales of our medical students are significantly elevated compared to the normative Austrian values. This is also in accordance with international studies which described similar findings in medical students [10, 27, 30, 35–37]. First year students, students in a relationship and male students showed the lowest frequency of burnout. The burnout rates in our study showed an increase during the study period from survey 1 to survey 2, while decreasing slightly at the end of the year (survey 3). In addition, the lowest rates of cynicism and emotional exhaustion were found within the beginner’s group, but increased to T2 and were found slightly reduced at T3. The overall burnout rate showed an almost linear increase from the beginners (year 2009) to the oldest students (2006).

In accordance with the Six Factor Model of Burnout by Maslach and Leiter [3], students in our study also had reduced scores of the AWLS in comparison to normative data, which is consistent with several international [29, 37, 38] findings. Furthermore, the subscales of the AWLS showed significant correlations with MBI, thus, documenting the impact of worklife factors on burnout. Maslach and Leiter [3] described burnout to be mainly a result of workplace deficits and interaction between work and employee, whereas Dyrbye et al. [39] for example could show that personal characteristics, personal life events, and learning environment influence the development of burnout separately. Thus, burnout in students has a similar origin as burnout in employees. In this study, students start the year with feeling for good fairness and high values, i.e., motivation, although they recognize the heavy workload at T1. But at T2 all factors of the AWLS are further diminished, at this time also the feeling of fairness and motivation are significantly reduced. At T3 a slight correction to better levels was detected. Heavy workload and the combination with lack of control, to less reward give the feeling of unfairness and reduce the possibility of building a community with support. Furthermore, this leads to feelings of unfairness and reduced motivation [19]. The temporal increase of burnout symptoms from T1 to T2 and the consecutive decrease over the summer holidays (T3) underlines these results.

On the personal side, Voltmer et al. [40] utilized cluster analysis in order to describe four different patterns of behavior in medical students that contributed to the development of burnout to various degrees. Pattern G (Health): high, but not too high engagement, high resilience, and a positive feeling of life; pattern S (care): low engagement, good resilience, and high satisfaction; pattern A (demand): low engagement, low resilience, and reduced satisfaction and pattern B (exhaustion): low engagement, strong resigned feelings, and very low satisfaction. Pattern G has a very low contribution to burnout, pattern B the highest. In addition, these patterns change over time with the exception of pattern B. If students start with this pattern [41], this pattern seems to be very stable. Dunn et al. conceptualized a model of burnout pathogenesis and prevention, the Model of Student Well-Being [42]. Factors leading to burnout in this model are stress, internal conflicts, time, and energy demands. Resilient factors according to this model are psychosocial support, social and healthy activities, mentorship, and intellectual stimulation. Personal factors in our study—such as perceived fairness, reward, and community as well as perceived external control—were also significantly reduced in comparison to normative data.

Others see stress as the central factor causing distress and exhaustion. Stress comes from a high workload (e.g., information to be learned), reduced time and control, exams and clinical procedures (such as dissection), low support from family, friends, colleagues and academic staff. Together with difficulties to relax, sleep deprivation, and feeling guilty, this results in a toxic combination that can lead to burnout symptoms and psychiatric disorders. We were not able to examine life events in our study, but we found that factors concerning academic work are highly correlated with burnout: workload, lack of support, and the other factors of the Six Factor Model.

### Limitations

As this was a naturalistic study, we used no control group design, which could be the next step to compare burnout rates of medial students to other faculties. A further limitation was the partly insufficient participation of students, which forced us to repeat part of the assessment two times. As there were no significant differences between the groups, we were happy to integrate them into the respective time points of the surveys. Another limitation is the lack of psychological, personal data of the students and their correlation to burnout measures; thus, we could mainly consider the workplace situation of the medical students. With respect to the statistical analysis, some might consider the absence of the Bonferroni correction for multiple testing to be a limitation of this study. This however is intentional, as it leads to a considerable reduction of statistical power to detect possible effects of explanatory variables on burnout [43].

## Conclusion

Burnout symptoms must to be taken into consideration in academic learning and teaching institutions. Burnout has to be differentiated into clinical and nonclinical presentations and services have to distinguish and help both groups. Several possibilities to reduce burnout have been described, enhancing student wellbeing by a personal [43] or institutional approach [42, 44, 45], thus, working with the proposed model. Furthermore, a combination of reduced or better balanced workload and good academic and psychological support, clear rules for fair exam, and possibilities to enhance a good supportive community will help students to cope better with the stressful situation of studying. For clinical cases relatively brief, individually focused, and mindfulness based interventions may be effective [1]. Others suggested to establish educational-participatory programs [46], coaching programs [47], or improving the relationship between students and academic staff [48], or by creating inspiring and authentic teaching [49]. Further suggestions are to provide mentoring programs [50] or implement communication and stress management trainings for medical students [51, 52] as was the case in Salzburg. In 2009 a new course was introduced, “Social Competence and Professionalism”, which lasts from the Beginners Seminar to the Closing-Camp at the end of the study period and also includes stress management and mindfulness training for medical students.

Therefore, a minimum requirement for medical schools according to our study and the literature cited is to screen for burnout and adapt the curriculum to preventive measures of stress as well as offering counseling and courses regarding mindfulness and relaxation to their medical students. Furthermore, vivid and modern didactic presentations (for example, “group puzzle”, “flipped classroom”), respect, and personal support should be provided by each university teacher.
